# Polylactic Acid/Lignin Composites: A Review

**DOI:** 10.3390/polym15132807

**Published:** 2023-06-25

**Authors:** Kang Shi, Guoshuai Liu, Hui Sun, Yunxuan Weng

**Affiliations:** 1 College of Chemistry and Materials Engineering, Beijing Technology and Business University, Beijing 100048, China; 2030402057@st.btbu.edu.cn (K.S.); 18838935071@163.com (G.L.); 2 Beijing Key Laboratory of Quality Evaluation Technology for Hygiene and Safety of Plastics, Beijing Technology and Business University, Beijing 100048, China

**Keywords:** poly (lactic acid), lignin, mechanical properties, composites

## Abstract

With the gradual depletion of petroleum resources and the increasing global awareness of environmental protection, biodegradable plastics are receiving more and more attention as a green substitute for traditional petroleum-based plastics. Poly (lactic acid) is considered to be the most promising biodegradable material because of its excellent biodegradability, biocompatibility, and good processability. However, the brittleness and high cost limit its application in more fields. Lignin, as the second largest renewable biopolymer in nature after cellulose, is not only rich in reserves and low in cost, but it also has an excellent UV barrier, antioxidant activity, and rigidity. The molecular structure of lignin contains a large number of functional groups, which are easy to endow with new functions by chemical modification. Currently, lignin is mostly treated as waste in industry, and the value-added utilization is insufficient. The combination of lignin and poly (lactic acid) can on the one hand solve the problems of the high cost of PLA and less efficient utilization of lignin; on the other hand, the utilization of lignocellulosic biomass in compounding with biodegradable synthetic polymers is expected to afford high-performance wholly green polymer composites. This mini-review summarizes the latest research achievements of poly (lactic acid)/lignin composites. Emphasis was put on the influence of lignin on the mechanical properties of its composite with poly (lactic acid), as well as the compatibility of the two components. Future research on these green composites is also prospected.

## 1. Introduction

In recent years, in light of the decreasing supply of petroleum resources and the environmental pollution caused by the extensive use of petroleum-based plastics, the development and preparation of biodegradable plastics as a sustainable substitute for petroleum-based plastics has attracted intensive research attention [1,2,3]. However, there is plenty of room for the development of biobased and biodegradable polymers. With regard to polymer from biomass, statistics found that only 1% of the 368 million tons of plastics produced each year originates from biomass [4].

### 1.1. Poly (Lactic Acid)

**Figure 1:**
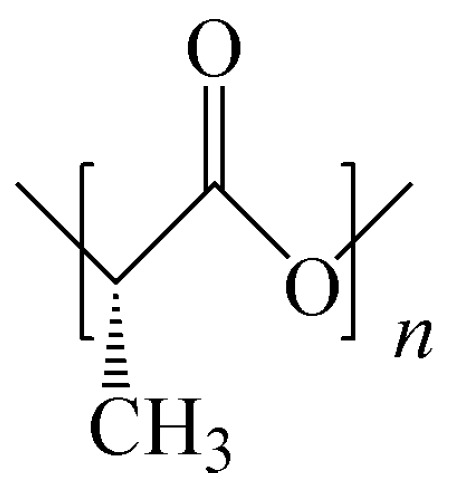
The structure of polylactic acid.

**Figure 2:**
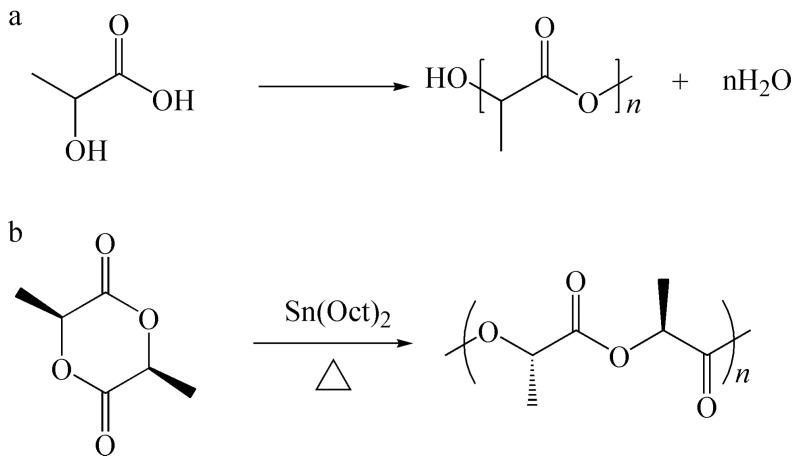
Synthetic pathway of polylactic acid. (**a**) Polymerization of lactic acid; (**b**) ring-opening polymerization of lactide.

Poly (lactic acid) (PLA) is the most promising biodegradable material at present, and its structure is shown in Figure 1 [5,6]. The polymerization of PLA can be divided into two types: the direct condensation polymerization of lactic acid monomers and the ring-opening polymerization of cyclic lactide (Figure 2). These two monomers can be obtained via the fermentation of renewable biomass such as starch and sugarcane. Therefore, PLA has excellent biocompatibility.

In addition to its inherent biocompatibility characteristics, compared with other aliphatic polyesters, PLA also has the following characteristics: (1) its tensile strength can reach 50–70 MPa and its shrinkage is low [7]; (2) it can be processed by a series of processing methods, such as injection molding, film extrusion, fiber spinning, etc., in the convenient temperature range of 170–230 °C. [8,9]; (3) less energy is needed in the production of PLA, 25–55% of energy reduction compared with other petroleum-based polymers [10]. Owing to these advantages, PLA is widely used in various fields, such as 3D printing [11,12], food packaging [13,14], biomaterials [15,16], adhesives [17,18], and so on.

However, one drawback associated with the rigidity of the main chain structure of this polymer is its brittleness and low elongation at break (4–6%) [19,20]. To overcome this drawback, petroleum-based derivatives such as polyethylene or chain extenders were blended to toughen PLA [21,22,23]. However, because most of these modifiers are non-degradable, their addition deteriorates the inherent biodegradability of PLA and conflicts with the concept of sustainable development. For the purpose of enhancing performance and maintaining its inherent biodegradability, modification using biomass macromolecules is considered to be a desirable choice.

### 1.2. Lignin

**Figure 3:**
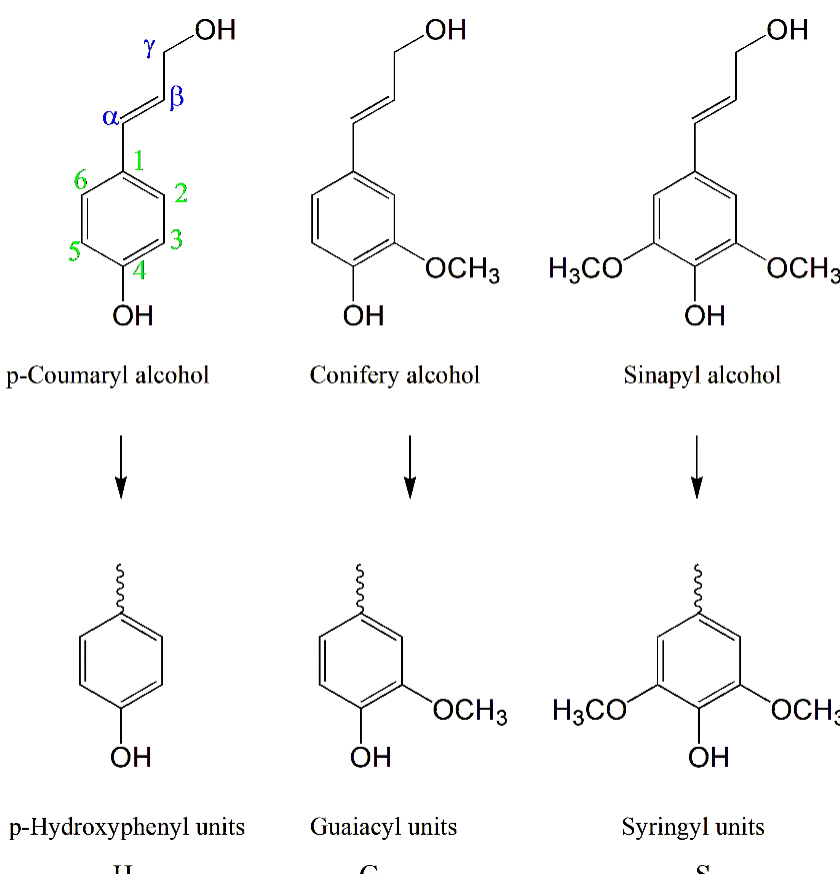
Structural formulas of three phenylpropane units of lignin.

Lignin is the second largest renewable bioresource after cellulose in the plant world. Lignin has an amorphous three-dimensional network structure consisting of *p*-hydroxyphenyl alcohol (H), guaiacyl (G), and syringyl (S) phenylpropane units (Figure 3) with a carbon–carbon bond and an ether bond [24,25].

The composition and content of lignin depend on its source. The proportion of lignin in hardwood is 20–25%, which is mainly composed of G and S units; the lignin content in coniferous wood is 25–35%, which is composed of a large number of G units and a small number of H units; the proportion of lignin in grass plants is 15–25%, and all three kinds of phenylpropane units exist [26,27].

Natural lignin is stable and insoluble in any reagents. The lignin used at present was obtained via separation and extraction from lignocellulose using physical or chemical methods. The properties of the obtained lignin vary with different extraction methods. The extraction methods of lignin can be divided into two categories: sulfur-containing and sulfur-free [28]. Table 1 outlines the common methods for the extraction of lignin.

Lignin contains a large number of active functional groups such as methoxy, phenolic hydroxyl, alcohol hydroxyl, and carbonyl groups, which can be conveniently used for modification by chemical reactions. Lignin also has the advantages of low cost, abundant reserves, good biodegradability, and antioxidant activity, which give this biopolymer huge application potential [33,34]. However, lignin is usually regarded as industrial waste and is only used in low-value applications. More than 50 million tons of lignin is produced each year, 98% of which is used for combustion and power generation, and only 2% is used for fillers, dispersants, and adhesives [35].

As an attempt in the modification of PLA using biomass, lignin was also used in combination with PLA. The combination of lignin and PLA offers a way for the preparation of wholly green PLA-based composites with reduced cost and enhanced properties, achieving the variolization of lignin.

In recent years, research on composites based on PLA and biopolymers mainly started from a broad direction [36,37]. The modification of biopolymers and the effects on the overall performance (mechanical, thermal, barrier, etc.) of PLA remain a challenge.

This mini-review summarizes the research progress of PLA–lignin composites with an emphasis on the effect of lignin on the mechanical properties of PLA and its compatibility aspects.

## 2. Research Progress of Polylactic Acid-Lignin Composites

### 2.1. Physical Blending

Because of its high carbon content, low density, and hardness, lignin (LG) is generally considered for the purpose of improving the mechanical properties of PLA. However, it was found that the addition of unmodified LG to PLA is prone to self-aggregation, leading to poor compatibility, and thus poor tensile strength and elongation at break [38]. The mechanical properties of some PLA–LG composites are shown in Table 2.

Anwer et al. [39] added kraft lignin (5%, 10%, and 15%) to PLA for the preparation of PLA–LG composites and studied the effect of LG on the mechanical and crystallization properties of PLA. They found that the tensile strength of the composites decreased with the increase in LG content, with a maximum decrease of 21%. The sharp decrease in tensile strength may be caused by stress concentration or low stress transfer efficiency. DSC measurement found that LG had an inhibitory effect on the crystallization of the composites. At a heating and cooling rate of 5 °C/min, the cold crystallization peak of the composites widened and moved to high temperature with the increase in LG content. This means that the delayed effect of LG as the second relative PLA crystallization is greater than its role as a heterogeneous nucleation site, resulting in the weakening of PLA’s crystallization ability.

Spiridon et al. [40] studied the effect of organosolv lignin (LO) addition on the mechanical properties of the PLA–LO composite. At 7% LO loading, the tensile strength of the composite was 48.76 MPa, lower than that of pure PLA (58.76 MPa), corresponding to a decrease of 17%. The decrease in tensile strength is due to the low adhesion of the interface between LO and PLA, and the stress cannot be effectively transferred to the matrix, which can easily cause stress concentration. The elongation at break also decreased from 6.0% to 2.5%, which may be due to the hydrogen bond and polarity interaction between PLA and LO, which limits the ductile flow of the polymer segment. When the addition of LO was further increased, a phenomenon different from that of Anwer [39] was observed. At 15% loading, the tensile strength and elongation at break of the material were significantly increased, and the tensile strength reached 60.23 MPa, even higher than that of pure PLA. This phenomenon can be attributed to the plasticizing effect of LO at high concentrations, which reduces the probability of the uneven aggregation and dispersion of particles at high concentrations. It was also found that LO acted as a rigid filler, and the elastic modulus and impact strength of the composites were improved to a certain extent with the addition of LO.

Pawale et al. [41] directly added commercial alkali lignin (CAL) to PLA and studied the effect of CAL on the mechanical and crystallization properties of PLA. With the addition of 10% CAL, the tensile strength and elongation at break of the composites decreased by 82% and 79%, respectively, when compared to pure PLA. Similar phenomena were also observed in two studies by Gordobil [44,45]. The significant decrease in tensile strength may be related to the non-uniform dispersion of LG in the PLA matrix, the fracture of the C-O-C bond, and microcracks caused by stress concentration [46]. A DSC study found that the addition of LG hindered the crystallization of the composites. With the increase in LG content, the *T*_cc_ of the composites moves to high temperature, from 104.32 °C to 108.63 °C, indicating that the energy required for the crystallization of the composites becomes higher and the crystallization becomes more difficult.

It is known that the size of the filler has a great influence on its dispersion in the matrix and the interface interaction between the filler and the matrix. Nanoparticles usually show different morphology and properties from micron particles because of their nanometer size, superior specific surface area, and good mechanical properties. The introduction of nanoparticles into green macromolecular composites is also one of the important methods to develop sustainable green composites. In recent years, many studies have focused on lignin nanoparticle–PLA composites.

Yang et al. [42] studied the effects of lignin nanoparticles (LNPs) on the mechanical, thermal, and crystallization properties of the composites. The results show that at 1% LNP loading, LNPs were uniformly dispersed in the PLA matrix. LNPs asheterogeneous nucleation sites promoted the nucleation of PLA, and the crystallinity of the composite was increased from 15.0% to 22.5%. The tensile strength increased from 44.3 MPa of pure PLA to 48.7 MPa. This is due to the uniform distribution of LNP in PLA, so that the stress can be transferred effectively in PLA. The elongation at break increased from 16.8% to 26.7%, which is contrary to that of many composites containing rigid nanoparticles, which may be related to the special chemical structure of LNPs. The large number of active functional groups present in LNPs promoted the interaction between PLA and LNPs, resulting in an increase in elongation at break. Further increasing the addition of LNPs reduced the tensile strength of the composites. This common observation is mainly because LNPs are prone to self-aggregation at high contents, thus preventing the formation of a long-range continuous phase of PLA [47]. The elongation at break was further increased to 66.2%. This phenomenon may be related to the presence of a large number of functional groups (carbonyl, hydroxyl, etc.) in LNPs. These structures significantly improve the hydrogen bonding interaction between LNPs and PLA, thus playing a toughening effect [47,48]. However, because of the self-aggregation of LNPs at high contents, the crystallinity of the composites decreased to 17.4%. It was also found that the thermal stability of the composites rose gradually with the increase in the content of LNPs, and the initial decomposition temperature of the composites increased from 259 °C to 274.7 °C of pure PLA, which can be attributed to the good thermal stability of LNPs itself.

Cavallo et al. [43] added LNP to PLA and studied the mechanical and UV absorption properties of the composite films. It was found that the mechanical properties of the films declined continuously with the addition of LNP, which may be related to the uneven dispersion of LNP in PLA. Because of the phenolic hydroxyl groups and other chromophores, LNP has excellent UV absorption properties [49]. Therefore, the UV absorption properties of the composite films were improved with the addition of LNP. However, the transparency of the film decreased with the addition. This is mainly due to two reasons: on the one hand, LNP of high content is easy to aggregate; on the other hand, LNP itself is rich in chromogenic groups, which will decrease the transparency of the polymer.

### 2.2. Chemical Modification

LG directly added to PLA is prone to self-aggregation because it is rich in hydroxyl groups. The aggregation affects the stress transfer and leads to a decline in the mechanical properties of PLA–LG composites. In order to overcome this deficiency, LG can be chemically modified to weaken the hydroxyl concentration and reduce its polarity, so as to improve its compatibility with PLA. The commonly used chemical modification methods included esterification, acetylation, and graft copolymerization, which are introduced in Table 3.

It is found that the compatibility of modified LG and PLA is greatly improved, which has a positive effect on the mechanical properties of the composites. The relevant mechanical data of some composites are shown in Table 4.

#### 2.2.1. Esterification Modification of Lignin

**Figure 4:**
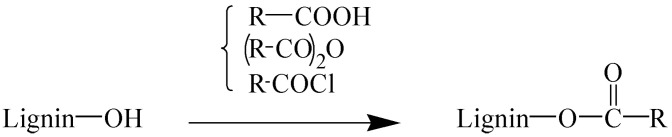
Esterification modification of lignin.

The esterification reaction is mainly carried out via the reaction of esterification reagents such as anhydride and acyl chloride with hydroxyl groups in LG (Figure 4). On the one hand, the reaction reduces the concentration of hydroxyl in LG, improves the hydrophobicity of LG, and improves the compatibility with PLA. On the other hand, esterification can increase the chain mobility of LG, reduce the glass transition temperature of LG, and increase its fluidity, which is helpful for improving the processability of the composites.

Park et al. [50] esterified LG with palmitoyl chloride and prepared composite films ELG–PLA via solution blending esterified lignin (ELG) and PLA. It was found that esterification decreased the content of hydroxyl and the strength of the hydrogen bond in LG. The increased chain mobility of ELG decreased *T*_g_; the decline in hydrogen bond strength rose with the increase in the esterification degree. In terms of mechanical properties, owing to the uniform distribution of ELG, and thus better compatibility, ELG–PLA composite film has a higher tensile strength and elastic modulus than LG–PLA.

Hong et al. [51] simply esterified LG with maleic anhydride, then melt blended with PLA to prepare composites. It was found that the interfacial adhesion of esterified LG and PLA was improved by hydrogen bonding. Compared with unmodified LG, ELG–PLA composite showed higher tensile strength.

Yan et al. [52] esterified LG with succinic anhydride and then compounded it with PLA. This hydrophobic modification of LG improved the interfacial compatibility of the composite. Besides good thermal stability, the mechanical properties of the composites were significantly enhanced, with the elastic modulus and tensile strength increased by 19.5% and 18.4%, respectively. At the same time, the composite showed antibacterial effects; the antibacterial efficiency against *Staphylococcus aureus* was up to 95%, indicating the potential for applications in the field of medical treatment and food packaging.

#### 2.2.2. Acetylation Modification of Lignin

Acetylation changes LG from hydrophilic to hydrophobic by replacing hydrophilic hydroxyl groups in LG. It can effectively reduce the hydrogen bond strength of LG, greatly enhances the compatibility of LG and PLA, and significantly improves the mechanical properties of the composites.

Gordobil et al. [45] acetylated LG and found that acetylated lignin (ALG) has lower *T*_g_ and higher hydrophobicity than LG. This is due to the fact that the replacement of hydroxyl with acetyl groups of LG reduced the concentration of hydroxyl groups in LG and weakened the hydrogen bonding interaction in LG molecules. As a result, better hydrophobicity and a faster chain transfer rate were obtained. When ALG was compounded with PLA, the tensile strength of the composite decreased slightly at low contents (<5%). The reduction is much less than that of LG–PLA, which can be attributed to the better interaction and better compatibility between ALG and PLA. With the addition of a low content of ALG, the elongation at break increased significantly, and the composite showed better ductility than pure PLA. This may be due to the acetylation; that is, acetyl played a role similar to that of a plasticizer [62].

Kim et al. [53] blended virgin lignin and ALG with PLA to prepare composites. It was found that the average size of LG in PLA increased with the increase in content (1%, 5%, and 10%), from 17.1 μm to 22.4 μm. The same phenomenon was observed in ALG, but its average size was much smaller than that of LG, only from 11.2 μm to 12.7 μm. This observation can be explained by the decrease in hydrogen bond strength due to the more uniform dispersion of ALG in PLA with fewer large aggregates. This phenomenon will have a positive effect on the mechanical properties of the composites. The UV-blocking properties of the composites were evaluated using a UV spectrophotometer. It was found that both LG–PLA and ALG–PLA composites had excellent UV-blocking properties. However, the UV-blocking properties of LG–PLA composites are lower than those of ALG–PLA, which is related to their compatibility with PLA.

**Figure 5:**
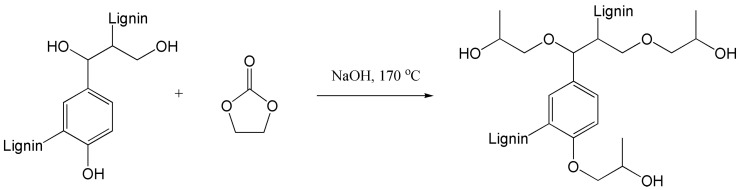
Hydroxypropylation of lignin [54].

Eakkimuthu et al. [54] improved the plasticity and fluidity of lignin using a hydroxypropylation reaction (Figure 5) and converted kraft lignin (KL) into oxypropylated kraft lignin (OPKL) with aliphatic groups at the end, so as to improve the compatibility between LG and PLA. By observing the cross-section of the composite, it was found that the addition of OPKL enhanced the smoothness of the cross-section of the composite and improved its compatibility in the PLA matrix, leading to the increase in the plasticity of the composite. Because most of the hydroxyl groups in KL were removed by hydroxypropylation, the strength of hydrogen bonding in KL decreased, and the free volume of the polymer system increased. As a result, the *T*_g_ of OPKL decreased from 95 °C to 84 °C. In addition, the introduction of OPKL reduced the *T*_cc_ value of the composites and promoted the heterogeneous nucleation of the composites. In terms of mechanical properties, with the addition of 5% OPKL, the composite has a tensile strength similar to that of PLA, about 39.6 MPa, and an elongation at break of 24.6%, increased by nearly 45% compared with that of PLA (17%). This phenomenon may be explained by low-molecular-weight lignin which played a role similar to a plasticizer. With the addition of OPKL, the mechanical properties showed a downward trend. This was because the increase in lignin led to more stress concentration [63]. Through the overall migration activity experiment, it was also found that the mobility of the composites with less than 10% OPKL content was maintained at 8.5 mg/kg, much lower than the total migration limit of 60 mg/kg stipulated in the Chinese national standard. Therefore, it can be considered that the composite has good application prospects in the field of food packaging.

#### 2.2.3. Modification of Lignin by Graft Copolymerization

Graft copolymerization was proved to be the most effective method of all modifying approaches. Various functional monomers can be introduced onto lignin via graft copolymerization. Examples of graft polymerization strategies included ordinary free radical polymerization, ring-opening polymerization, and living free radical polymerization.

##### Ordinary Free Radical Polymerization

Ordinary radical polymerization mainly includes three stages: chain initiation, chain transfer, and chain termination. It mainly has the following advantages: (1) the applicability of a wide range of monomers [64,65]; (2) the feasibility of preparing copolymers with any number of monomers; (3) the availability of multiple polymerization methods; (4) and being simple and low-cost.

Xin et al. [55] modified PLA–LG composites with dicumyl peroxide (DCP) or maleic anhydride (MA)/dibenzoyl peroxide (BPO), respectively. DCP or BPO extracts hydrogen ions from the molecular chain of PLA and the hydroxyl site of LG to produce macromolecular free radicals. PLA radicals and lignin macromolecular radicals combine to form C−C covalent bond, which enhances the interaction. Alternatively, in the presence of MA/BPO, MA is grafted directly to the PLA chain and then reacts with the hydroxyl groups in LG to form ester bonds, thus improving the compatibility of LG and PLA.

It was found that the mechanical properties of PLA–LG composites reached the maximum at 4% LG loading; the tensile strength and modulus increased from 48.5 MPa and 2.7 GPa (pure PLA) to 59.9 MPa and 3.2 GPa, respectively. This result can be attributed to the highly concentrated benzene ring structure of LG [55,66]. Additionally, it was also supposed to be related to the special chemical structure of lignin, bearing a lot of functional groups (carbonyl, phenolic, or aliphatic hydroxyls, carboxyl), which intensively contributed to the interactions (like hydrogen bonding) between the PLA matrix and LG [47]. At the same time, the elongation at break also increased from 3.4% to 3.8%. The slight increase in elongation at break is also closely related to the special structure of lignin (the benzene ring is connected to the ether bond as a plasticizer).

The tensile strength and modulus of the composites modified with DCP were significantly improved, up to 69.8 MPa (0.2% DCP) and 4.5 GPa (2.0% DCP), respectively. However, the elongation at break began to decline at DCP contents above 0.4%. On the one hand, this phenomenon is related to the agglomeration of DCP at high concentrations; on the other hand, DCP enhanced the interaction between LG and PLA, resulting in shorter C−C covalent bonds, which hindered the movement of the polymer chain. For the composites treated by MA–BPO, the tensile strength and elongation at break reached the highest, i.e., 71.6 MPa and 5.0%, respectively, at 0.4% MA addition. The former can be attributed to the strong interaction between PLA and LG in the presence of MA–BPO. The latter is mainly due to the high toughness of MA formed in the composites.

**Figure 6:**
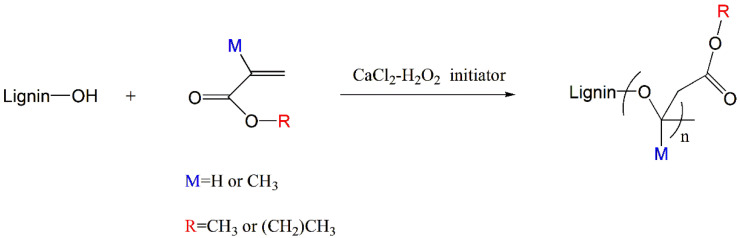
Synthesis of lignin graft copolymer via free radical polymerization.

Zong et al. [56] reported the graft polymerization of different acrylate monomers (methyl methacrylate PMMA, *n*-butyl methacrylate PBMA) onto acetic acid lignin (AAL) via ordinary free radical polymerization (Figure 6. The modified lignin was then melted with PLA for the preparation of the composites. It was found that the *T*_g_ of AAL was 125.2 °C, which decreased to 110 °C of AAL-g-PMMA and 32.1 °C of AAL-g-PBMA, respectively, after grafting. The contact angle increased from 44° to 105° and 90°, indicating that the concentration of hydroxyl in AAL decreased and the hydrophobicity of the grafted product was improved after the introduction of acrylate monomer. An unexpected phenomenon in the mechanical properties of the composites was observed. At a 10% AAL-g-PBMA loading, the composite achieved an elongation at break of 28%, which is more than three times higher than that of pure PLA and 6.7% of AAL. This is probably due to the long side chain of PBMA, which makes AAL-g-PBMA extremely flexible, resulting in good interfacial adhesion to PLA and thus an excellent toughening effect on PLA [67]. The possibility of the composite as a UV barrier agent was also evaluated. It was found that the composite had excellent UV barrier performance. The UV barrier rate in the UV-C (100–280 nm) and UV-B (280–315 nm) areas is close to 100%, and the UV barrier rate in the 360 nm area is more than 80%.

As a continuation of Zong’s work, Sun et al. [57] further functionalized LG by chemically grafting biological acrylic acid monomers of dodecyl methacrylate (LMA) and tetrahydrofurfuryl methacrylate (THFMA). Then, the lignin graft copolymer was melted with PLA. PLA is a typical brittle material with a tensile strength of 63.5 MPa, an elongation at break of 12%, and a fracture toughness of as low as 4.4 MJ/m^3^. With the addition of lignin graft copolymer, the elongation at break and fracture toughness of the composites experience a V-type change with increasing lignin contents, which were still higher than those of pure PLA. At 20% graft copolymer loading, the elongation at break reached the highest (204%), 17 times that of PLA. This can be attributed to the excellent flexibility of the graft copolymer and the strong interfacial adhesion to PLA. Studies have found that many voids and PLA filaments were observed in the cross-section of the composite; a large number of deformed lignin phases were found near the voids, and some of them were even pulled out from the PLA. The formation of voids and the pullout of the lignin phase can not only induce the plastic deformation of PLA but also contribute to the energy dissipation in the tensile process, leading to the excellent ductility and fracture toughness of the composites.

##### Ring-Opening Polymerization

Ring-opening polymerization is a reaction in which cyclic compound monomers are transformed into linear polymers by ring-opening addition. Ring-opening polymerization shares the advantages of mild reaction conditions, few side reactions, and the convenience to obtain a higher molecular weight.

**Figure 7:**
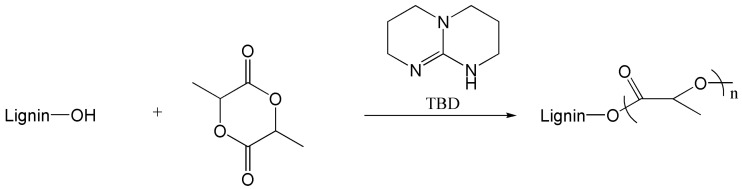
Synthesis of lignin graft copolymer via ring-opening polymerization [58].

Chung et al. [58] initiated solvent-free ring-opening grafting polymerization (see Figure 7) to obtain lignin-g-PLA copolymers for the preparation of composites with PLA. After grafting PLA, the polarity of LG changed from hydrophilic to hydrophobic, which weakened the self-aggregation of lignin, leading to excellent compatibility between the lignin-graft copolymer and PLA. At 0.9% loading of lignin-g-PLA, the tensile strength and elongation at break of the composite increased by 16% and 9%, respectively. With further addition, the mechanical properties decreased. This result showed that a small number of lignin-g-PLA copolymers can effectively improve the mechanical properties of PLA. It was found that with a small amount of lignin graft copolymer loading (0.9%), the composites showed excellent UV barrier properties, which could block almost all UV-C and UV-B, as well as more than half of UV-A.

Boarino et al. [59] prepared PLA-g-lignin nanoparticles with (PLA-g-LNPs)–PLA thin films and studied the effect of PLA-g-LNPs on the properties of the films. In order to prevent the aggregation of LNPs in PLA, Boarino grafted lactide onto LNPs using ring-opening polymerization. At 10% loading, PLA-g-LNPs could still be uniformly dispersed in PLA. Due to the good dispersion of PLA-g-LNPs, the PLA-g-LNP–PLA composite films not only have stronger UV absorption and transparency in the visible region but also greatly improve the oxidation resistance of the films. The elongation at break of the composite samples increased when 1 wt.% filler was incorporated and even further upon increasing the lignin content to 5 wt.% for the PLA–lignin composite and the PLA-g-LNP–PLA composite. Further increasing the lignin content to 10%, resulted in a decrease in elongation at break for the PLA–lignin composite, probably due to phase separation and the low cohesion between lignin and PLA. In contrast, the PLA-g-LNP–PLA composite showed a further increase in elongation at break upon increasing the PLA-g-LNP content from 5 to 10 wt.%. This is attributed to the presence of the grafted PLA chains that act as compatibilizers.

#### 2.2.4. Further Options of Lignin Modification

Living radical polymerization includes atom transfer radical polymerization (ATRP) and reversible addition–fragmentation chain transfer (RAFT). This method can be used to control the molecular weight of polymers and to prepare functional polymers with regular structures. In recent years, RAFT has been widely used in various fields [68].

ATRP is a green free radical polymerization technology, which requires only a small amount of catalyst and is suitable for industrial scale-up.

**Figure 8:**
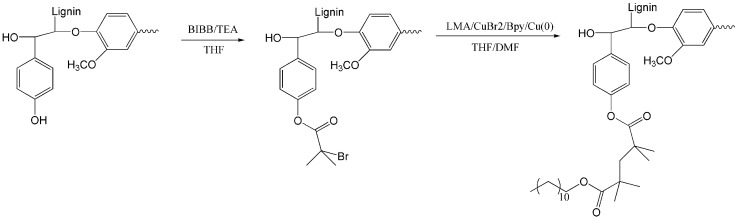
Synthesis of lignin graft copolymer using ATRP [60].

**Figure 9:**
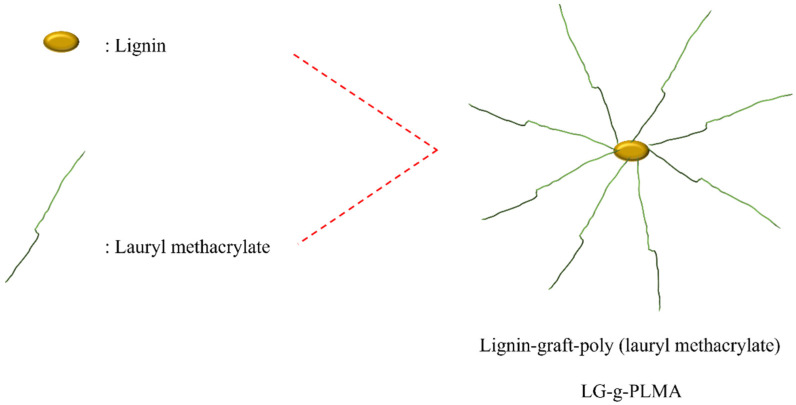
The structure of LG-g-PLMA [60].

Wu et al. [60] successfully synthesized LG-g-PLMA lignin-based copolymers by introducing biological monomer lauryl methacrylate (LMA) into the molecular chain of organic solvent lignin using the ATRP method (Figure 8). The copolymers with different lignin contents were controllably synthesized by changing the ratio of the LMA to the macromolecular initiator. It was found that the relative molecular weight of the copolymer decreased with the increase in macromolecular initiator content. This is due to the fact that with a sufficient macromolecular initiator, the number of grafted PLMA molecular chains increased, but the length decreased accordingly. As a result, a large number of PLMA short-branched chains formed on the copolymer, leading to a decrease in relative molecular weight [69]. Atomic force microscope (AFM) observation found that lignin showed a spherical dot morphology on which the copolymer LG-g-PLMA exhibited many vertical nano-needle-like structures, and the number increased with the increasing lignin content, and they were uniformly distributed in the dots. Therefore, a possible core–shell structure is proposed for LG-g-PLMA (Figure 9). As shown in Figure 9, PLMA is the shell and lignin is the core, with multiple flexible PLMA chains grafted onto the rigid lignin core to form a relatively independent micro-component, which is then aggregated by the interaction with PLMA. Because LMA has good hydrophobicity, LG-g-LMA will have good compatibility with PLA and other polyester and is expected to be used as a functional additive in polyester.

Bao and coworkers [61] used horseradish peroxidase (HPR)/acetylacetone (ACAC)/hydrogen peroxide (H_2_O_2_) as a catalytic system for the RAFT polymerization of different vinyl monomers (PAM, PBA) on lignin. Different from the traditional lignin grafting method, this method avoided the use of a chemical initiator in the synthesis of the lignin chain transfer agent. GPC studies found that the lignin-grafted copolymers using the RAFT method had higher *M*_n_ (41,553 and 88,946 g/mol) and narrow molecular weight distribution (PDI: 2.11, 1.82) compared with unmodified lignin (*M*_n_: 17,050 g/mol, PDI: 2.3). This method is instructive for the green synthesis of lignin-based graft copolymers. However, the application of the copolymer in PLA and other polyesters remains to be explored.

## 3. Conclusions and Outlook

Among industrial plastics, PLA is considered to be the most promising biodegradable material because of its excellent biocompatibility, good processability, and complete degradability (PLA breaks down to CO_2_ and H_2_O in the natural environment). However, its high price and poor toughness limit its further application. The compounding of LG and PLA cannot only reduce the cost of the composite but also give PLA excellent rigidity as well as optical and thermal stability.

Because of its large number of hydrogen bonds, LG has strong intermolecular and intramolecular forces, resulting in less satisfied dispersion in PLA, and thus a decrease in the mechanical properties of the composites. However, studies found that after modification, LG can be uniformly dispersed in PLA, and the toughness of the composites was greatly improved. Even a transition from brittleness to toughness was observed. Among the modification methods of LG, graft copolymerization was found to be the most effective for improving the compatibility of LG and PLA. By grafting various monomers, new properties can be introduced to LG and its composites.

In this regard, future research focusing on PLA–LG composites could include the following aspects: (1) the graft copolymerization of LG to create green and feasible grafting methods; (2) the improvement of the composites’ properties by modifying PLA and then compounding with LG; (3) taking advantage of the excellent UV barrier and thermal stability of LG to expand the application of composites in the field of packaging; and (4) fully develop the biological characteristics of PLA–LG composites and develop and research their applications in the field of biomedicine.

## Figures and Tables

**Table 1:** Classification of lignin extraction methods.

Extraction Method	Type of Lignin	Scale	Reaction Condition	Solubility	Ref.
Sulfur-containing	Kraft lignin	Industrial	170 °C, NaOH + Na_2_S	Alkali, organic solvents	[29]
	Lignosulfonate	Industrial	140 °C, SO_2_^+^Na^+^/Ca^+^/Mg^+^, NH_4_^+^	Water	[30]
Sulfur-free	Soda lignin	Industrial/Pilot	150–170 °C, NaOH	Alkali	[31]
	Organosolv lignin	Industrial/Pilot	90–210 °C, Organosolv	Wide range of organic solvents	[32]

**Table 2:** Mechanical properties of PLA–LG composites.

PLA	Type of LG *	LG Mass Fraction (%)	TS ** (MPa)	EB *** (%)	Ref.
3052D	KL	0	70.16	-	[39]
3052D	KL	15	55.24	-	[39]
2002D	LO	0	58.76	6.00	[40]
2002D	LO	7	48.76	2.50	[40]
3052D	CAL	0	66.31	4.34	[41]
3052D	CAL	10	11.88	0.90	[41]
3251D	LNPs	0	44.30	16.80	[42]
3251D	LNPs	1	48.70	26.70	[42]
3251D	LNP	0	49.92	8.23	[43]
3251D	LNP	3	53.65	3.35	[43]

* KL: kraft lignin; LO: organosolv lignin; CAL: commercial alkali lignin; LNPs: lignin nanoparticles from steam lignin by treatment with HCL; LNP: lignin nanoparticles from alkali lignin via treatment with HCL. ** TS: tensile strength. *** EB: elongation at break.

**Table 3:** Chemical modification of the lignin.

Modification Method *	Reaction Monomer **	Reaction Condition ***	Ref.
Esterification	Lignin, PC	THF, 65 °C, 48 h, N_2_	[50]
Esterification	Lignin, MA	DMF, 120 °C, 6 h	[51]
Esterification	Lignin, SAn	THF, 60 °C, 2 h	[52]
Acetylation	Lignin, AA, Py	Formamide, RT, 36 h	[45]
Acetylation	Lignin, AA, Py	RT, 24 h	[53]
Hydroxypropylation	Lignin, PPC	NaOH, 170 °C, 3 h	[54]
FRP	Lignin, MA	Melting compound	[55]
FRP	Lignin, MMA, BMA	DMSO, 50 °C, 24 h	[56]
FRP	Lignin, LMA, THFMA	DMSO, 50 °C, 24 h	[57]
ROP	Lignin, L-lactide	130 °C, 3.5 h, N_2_	[58]
ROP	LNP, DL-lactide	RT, 3 h, N_2_	[59]
ATRP	1. Lignin, TEA, BiBB 2. LMA, Bpy, CuBr_2_	1. THF, 65 °C, 48 h, N_2_ 2. THF,70 °C, 24 h, N_2_	[60]
RAFT	Lignin, DDMAT, HRP	Buffer (0.1 M, pH 7.0) 40 °C, 24 h	[61]

* FRP: free-radical polymerization; ROP: ring-opening polymerization; ATRP: atom transfer radical polymerization; RAFT: reversible addition–fragmentation chain transfer polymerization. ** PC: palmitoyl chloride; MA: maleic anhydride; SAn: succinic anhydride; Py: pyridine; PPC: propylene carbonate; MMA: methyl methacrylate; BMA: N-Butyl methacrylate; LMA: lauryl methacrylate; THFMA: tetrahydrofurfuryl methacrylate; TEA: triethylamine; BiBB: α-Bromoisobutyryl bromide; Bpy: 2,2-dipyridyl; DDMAT: 2-(Dodecylthiocarbonothioylthio)-2 methylpro-pionic acid: HRP: Horseradish peroxidase. *** THF: tetrahydrofuran; DMF: N, N-dimethylformamide; DMSO: dimethyl sulfoxide; RT: room temperature; Buffer: 20/80 (*v*/*v*) ethanol/phosphate.

**Table 4:** Mechanical properties of PLA–LG composites.

Sample *	TS (MPa)	EM ** (GPa)	EB (%)	Ref.
PLA/10%LG	36.6	0.26	1.3	[50]
PLA/10%ELG	42.3	0.31	1.1	[50]
PLA/20%LG	23	2.4	-	[51]
PLA/20%ELG	55	5.0	-	[51]
PLA/5%LG	43.5	0.22	1.9	[45]
PLA/5%ALG	44.5	0.26	3.9	[45]
PLA/5%LG	65.2	2.53	7.9	[53]
PLA/5%ALG	70.3	2.51	9.5	[53]
PLA/5%LG	33.6	-	4.6	[54]
PLA/5%OPKL	39.6	-	24.6	[54]
PLA/4%LG	59.9	3.2	3.8	[55]
PLA/4%LG/0.4%DCP	64.2	3.1	3.6	[55]
PLA–LG/0.4%MA	71.6	5.0	4.2	[55]
PLA/10%LG	59.2	1.1	6.7	[56]
PLA/10%LG-g-PBMA	61.5	0.9	28	[56]
PLA	63.5	2.4	12	[57]
PLA/20%MLG	40.9	2.46	204	[57]
PLA/10%LG	24.9	2.8	1.1	[59]
PLA/10%PLA-g-LNPs	23.5	1.9	8.5	[59]

* ELG: esterification lignin; ALG: acetylated lignin; OPKL: oxypropylated kraft lignin; DCP: dicumyl peroxide; MLG: LG-g-P(LMA-co-THFMA). ** EM: elastic modulus.

## Data Availability

Not applicable.
